
JOURNAL INFORMATION
==============================
NLM Title Abbreviation: ScientificWorldJournal
Journal ID: ScientificWorldJournal
NLM Journal ID: 101131163
Journal ID: TSWJ

The Scientific World Journal

ISSN: 2356-6140
EISSN: 1537-744X
Publisher: Wiley

ARTICLE INFORMATION
==============================
PMCID: PMC5917414
PMID: 16604255
DOI: 10.1100/tsw.2006.88
Article ID: 870965
Article version: 1
Subjects: Directions in Science

Proteoglycans are Differentially Altered in Muscular Dystrophies

Zanotti Simona
Mora Marina *
Division of Neuromuscular Diseases and Neuroimmunology, Istituto Nazionale Neurologico “C. Besta”, Via Temolo 4, 20126 Milan, Italy
*Marina Mora: mmora@istituto-besta.itAcademic Editor: Martin Goette

Publication date: 2006
Electronic publication date: 2006 Apr 4
Volume: 6
First page: 446
Last page: 448
Received 2006 Feb 27; Accepted 2006 Mar 27
Copyright: Copyright © 2006 Simona Zanotti and Marina Mora.
Copyright year: 2006
License: This is an open access article distributed under the Creative Commons Attribution License, which permits unrestricted use, distribution, and reproduction in any medium, provided the original work is properly cited.
License URL: https://creativecommons.org/licenses/by/3.0/

Keywords: proteoglycans, decorin, biglycan, fibrosis, connective tissue, proliferation, muscular dystrophy

==============================
Directions in Science
*TheScientificWorldJOURNAL* (2006) 6, 446–448
ISSN 1537-744X; DOI 10.1100/tsw.2006.88

TheScientificWorldJOURNAL
www.thescientificworld.com

### Proteoglycans are Differentially Altered in Muscular Dystrophies

Simona Zanotti and Marina Mora*

*Muscle Cell Biology Lab, Division of Neuromuscular Diseases and Neuroimmunology, Istituto Nazionale Neurologico "C. Besta", Via Temolo 4, 20126 Milano, Italy*

**E-mail**: szanotti@istituto-besta.it; mmora@istituto-besta.it

*Received February 27, 2006; Accepted March 27, 2006; Published April 4, 2006*

---

**KEYWORDS**: proteoglycans, decorin, biglycan, fibrosis, connective tissue, proliferation, muscular dystrophy

---

Muscular dystrophies are muscle diseases due to mutations in various genes that affect the stability and viability of muscle fibers. The common result is that the muscle fibers degenerate and die (necrosis), and although they regenerate to variable extents, the muscle tissue is progressively substituted by fibrotic connective tissue (fibrosis)[1].

These diseases may manifest at birth, in early infancy, or later, and progress with variable severity[1]. Although most forms are rare, some, like the severe Duchenne muscular dystrophy (DMD) that affects about 1 in 3500 newborn boys, are not infrequent.

In fibrosis, connective tissue structural proteins like collagens and laminins are laid down in the muscle[2]; however, the expression of regulatory factors such as cytokines (particularly transforming growth factor-β) and proteoglycans (which may have structural or regulatory functions) is also altered in the muscle. Transforming growth factor-β1 (TGF-β1) has been linked to fibrosis in kidney, liver, and lung diseases as well as in muscle diseases[3,4,5] and may actually be involved in stimulating the deposition of connective tissue proteins that ultimately make it impossible for muscle cells to regenerate, although this is still conjectural.

The paper by Zanotti and colleagues[6] aimed to better understand mechanisms of muscle fibrosis in various muscular dystrophies by investigating the expression of decorin and biglycan in relation to other molecules in muscle.

Biglycan and decorin are structurally similar, extracellularly located, small leucine-rich proteins known as proteoglycans because glycosaminoglycan carbohydrate chains are attached to the protein part[7]. Numerous functions of biglycan and decorin have been documented, most notably cell adhesion, matrix assembly, modulation of signal transduction, and interactions with matrix proteins, growth factors, and their receptors[8,9,10,11,12,13,14]. Functions of likely importance in muscle diseases include binding to collagens, binding to TGF-β1[15,16,17], and interaction (of biglycan) with α-dystroglycan[18], a component of the dystrophin-associated protein complex on the muscle cell membrane, which is often disrupted in muscular dystrophies.

Zanotti and colleagues determined the extent of fibrosis and found, as expected, that it was greater in all patient muscle than in healthy (control) muscle; however, in the severe diseases DMD and congenital muscular dystrophy 1A (MDC1A), the fibrosis was very extensive. They also found variations in the levels of the studied proteoglycans and of their mRNAs, in the different muscular dystrophies studied,

*Corresponding author.
©2006 with author.
Published by TheScientificWorld, Ltd.; www.thescientificworld.com probably reflecting the variable disruption of connective tissue organization that occurs in these diseases. Importantly, they found significantly lowered decorin levels in DMD and MDC1A in association with markedly high levels of TGF-β1. It seems that because of its low levels, decorin was unable to perform one of its normal functions, that of lowering the biological activity of TGF-β1[19,20,21]; the increased TGF-β1 levels were therefore worsening the fibrosis. The possible implication of this finding is that treating patients with decorin may reduce the proliferation of muscle fibrosis and slow the progression of the disease. Studies on the role of decorin in muscular dystrophies and its potential therapeutic value are continuing.

### REFERENCES

1. Engel, A.G., Yamamoto, M., Fishbeck, K.H. (1994) Muscular dystrophies. In *Myology*. 2nd ed. Engel, A.G. and Franzini-Armstrong, C., Eds. McGraw-Hill, New York. pp. 1130–1187.
2. Stephens, H.R., Duance, V.C., Dunn, M.J., Bailey, A.J., and Dubowitz, V. (1982) Collagen types in neuromuscular diseases. *J. Neurol. Sci.* **53**, 45–62.
3. Coker, R.K. and Laurent, G.J. (1998) Pulmonary fibrosis: cytokines in the balance. *Eur. Respir. J.* **11**, 1218–1221.
4. Sime, P.J., Xing, Z., Graham, F.L., Csaky, K.G., and Gauldie, J. (1997) Adenovector-mediated gene transfer of active transforming growth factor-beta1 induces prolonged severe fibrosis in rat lung. *J. Clin. Invest*. **100**, 768–776.
5. Bernasconi, P., Torchiana, E., Confalonieri, P., Brugnoni, R., Barresi, R., Mora, M., Cornelio, F., Morandi, L., and Mantegazza, R. (1995) Expression of transforming growth factor β-1 in dystrophic patient muscles correlates with fibrosis. *J. Clin. Invest.* **96**, 1137–1144.
6. Zanotti, S., Negri, T., Cappelletti, C., Bernasconi, P., Canioni, E., Di Blasi, C., Pegoraro, E., Angelini, C., Ciscato, P., Prelle, A., Mantegazza, R., Morandi, L., and Mora, M. (2005) Decorin and biglycan expression is differentially altered in several muscular dystrophies. *Brain* **128**, 2546–2555
7. Krusius, T. and Ruoslahti, E. (1986) Primary structure of an extracellular matrix proteoglycan core protein deduced from cloned cDNA. *Proc. Natl. Acad. Sci. U. S. A.* **83**, 7683–7687.
8. Kobe, B. and Deisenhofer, J. (1994) The leucine rich repeat: a versatile binding motif. *Trends. Biochem. Sci.* **19**, 415–421.
9. Vogel, K.G., Paulsson, M., and Heinegard, D. (1984) Specific inhibition of type I and type II collagen fibrillogenesis by the small proteoglycan of tendon. *Biochem. J.* **223**, 587–597.
10. Winnemoller, M., Schmidt, G., and Kresse, H. (1991) Influence of decorin on fibroblast adhesion to fibronectin. *Eur. J. Cell Biol.* **54**, 10–17.
11. Bidanset, D.J., LeBaron, R., Rosenberg, L., Murphy-Ullrich, J.E., and Hook, M. (1992) Regulation of cell substrate adhesion: effects of small galactosaminoglycan-containing proteoglycans. *J. Cell Biol.* **118**, 1523–1531.
12. Santra, M., Eichstetter, I., and Iozzo, R.V. (2000) An anti-oncogenic role for decorin. Down-regulation of ErbB2 leads to growth suppression and cytodifferentiation of mammary carcinoma cells. *J. Biol. Chem.* **275**, 35153–35161.
13. De Luca, A., Santra, M., Baldi, A., Giordano, A., and Iozzo, R.V. (1996) Decorin-induced growth suppression is associated with up-regulation of p21, an inhibitor of cyclin-dependent kinases. *J. Biol. Chem.* **271**, 18961–18965.
14. Kinsella, M.G., Tsoi, C.K., Järveläinen, H.T., and Wight, T.N. (1997) Selective expression and processing of biglycan during migration of bovine aortic endothelial cells. The role of endogenous basic fibroblast growth factor. *J. Biol. Chem.* **272**, 318–325.
15. Yamaguchi, Y., Mann, D.M., and Rouslahti, E. (1990) Negative regulation of transforming growth factor-beta by the proteoglycan decorin. *Nature* **19**, 281–284.
16. Noble, N.A., Harper, J.R., and Border, W.A. (1992) In vivo interactions of TGF-beta and extracellular matrix. *Prog. Growth Factor Res.* **4**, 369–382.
17. Hildebrand, A., Romaris, M., Rasmussen, L.M., Heinegard, D., Twardzik, D.R., Border, W.A., and Ruoslahti, E. (1994) Interaction of the small interstitial proteoglycans biglycan, decorin and fibromodulin with transforming growth factor beta. *Biochem. J.* **302**, 527–534.
18. Bowe, M.A., Mendis, D.B., and Fallon, J.R. (2000) The small leucine-rich repeat proteoglycan biglycan binds to alpha-dystroglycan and is upregulated in dystrophic muscle. *J. Cell Biol.* **148**, 801–881.
19. Border, W.A., Noble, N.A., Yamamoto, T., Harper, J.R., Yamaguchi, Y., Pierschbacher, M.D., and Ruoslahti, E. (1992) Natural inhibitor of transforming growth factor-beta protects against scarring in experimental kidney disease. *Nature* **360**, 361–364.
20. Giri, S.N., Hyde, D.M., Bruan, R.K., Gaarde, W., Harper, J.R., and Pierschbacher, M.D. (1997) Antifibrotic effect of decorin in a bleomycin hamster model of lung fibrosis. *Biochem. Pharmacol.* **54**, 1205–1216.
21. Logan, A., Baird, A., and Berry, M. (1999) Decorin attenuates gliotic scar formation in the rat cerebral hemisphere. *Exp. Neurol.* **159**, 504–510.

**This article should be cited as follows:**

Zanotti, S. and Mora, M. (2006) Proteoglycans are differentially altered in muscular dystrophies. *TheScientificWorldJOURNAL* **6**, 446–448. DOI 10.1100/tsw.2006.88.

### BIOSKETCH

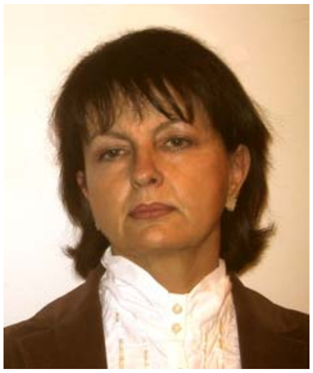

**Dr. Marina Mora** — After graduating in Biological Sciences at the University of Milano, Dr. Mora has been trained in muscle pathology at the Division of Neuromuscular Diseases of the Besta Institute in Milano where she was mainly involved in morphological studies on normal and pathological muscle of humans and animal models. She then received a postdoctoral fellowship at the Neuromuscular Disease Center of Mayo Clinic, U.S. (Dr. A.G. Engel, Director) where she was mainly involved in morphological ultrastructural studies of normal and pathological neuromuscular junction. Since then, Dr. Mora's studies have expanded from histopathology to *in vivo* and *in vitro* investigations on muscle cell growth and differentiation and, more recently, to genetic and molecular characterization of muscle diseases. Such studies have produced more than 70 papers that have appeared in international peer reviewed journals.

Dr. Mora is now Head of the Muscle Cell Biology Laboratory in the Department of Neuromuscular Diseases, National Neurological Institute "C. Besta", Milano, Italy and leader of a research group involved in molecular characterization of defects causing several muscular dystrophies and in studies on pathogenetic mechanisms responsible for such diseases.
